# NFE2L2/NRF2 silencing-inducible miR-206 targets c-MET/EGFR and suppresses BCRP/ABCG2 in cancer cells

**DOI:** 10.18632/oncotarget.22513

**Published:** 2017-11-18

**Authors:** Bo-Hyun Choi, Da Young Ryu, In-Geun Ryoo, Mi-Kyoung Kwak

**Affiliations:** ^1^ Department of Pharmacy and BK21 PLUS Team for Creative Leader Program for Pharmacomics-Based Future Pharmacy, Graduate School of The Catholic University of Korea, Bucheon, Gyeonggi-do 420-743, Republic of Korea; ^2^ College of Pharmacy, The Catholic University of Korea, Bucheon, Gyeonggi-do 420-743, Republic of Korea

**Keywords:** hepatocyte growth factor receptor (HGFR/c-MET), epidermal growth factor receptor (EGFR), NFE2L2/NRF2, BCRP/ABCG2, microRNA-206 (miR-206)

## Abstract

The nuclear factor (erythroid-derived 2)-like 2 (NFE2L2/NRF2) plays a critical role in the expression of multiple antioxidant and detoxifying enzymes. Herein, we provide evidence of the molecular links between NRF2 and oncogenic signaling hepatocyte growth factor receptor (HGFR/c-MET) and epidermal growth factor receptor (EGFR). Interfering RNA-induced stable inhibition of *NRF2* in ovarian carcinoma SKOV3 and renal carcinoma A498 reduced the levels of c-MET and EGFR. MicroRNA-206 (miR-206) that was increased in both *NRF2*-silenced cells was predicted as a dual regulator of c-MET and EGFR. As experimental evidence, miR-206 decreased c-MET and EGFR levels through a direct binding to the 3′-untranslated region of the *c-MET* and *EGFR* genes. The treatment of *NRF2-*knockdown cells with the miR-206 inhibitor could restore c-MET and EGFR levels. The miR-206-mediated c-MET/EGFR repression resulted in two outcomes. First, presumably through the inhibition of c-MET/EGFR-dependent cell proliferation, overexpression of miR-206 inhibited tumor growth in SKOV3-inoculated nude mice. Second, reduced c-MET/EGFR in *NRF2*-silenced cells affected breast cancer resistance protein (BCRP/ABCG2) levels. The pharmacological and genetic inhibition of c-MET or EGFR, as well as the miR-206 mimic treatment, repressed BCRP levels and increased cellular accumulation of doxorubicin. In line with these, treatment of *NRF2*-silenced SKOV3 with the miR-206 inhibitor elevated BCRP levels and consequently made these cells more resistant to doxorubicin treatment. Collectively, our results demonstrated that the *NRF2* silencing-inducible miR-206 targeted both c-MET and EGFR, and subsequently suppressed the BCRP level in cancer cells.

## INTRODUCTION

Dysregulation of receptor tyrosine kinases (RTKs), including hepatocyte growth factor receptor (HGFR/c-MET) and epidermal growth factor receptor (EGFR), has been implicated in cancer development and progression through a wide range of signaling pathways such as phosphatidylinositol 3-kinase (PI3K)-AKT and mitogen-activated protein kinase (MAPK) [1, 2]. Various types of cancers exhibit high levels of c-MET via gene amplification, overexpression, activating mutations, and increased autocrine or paracrine ligand-mediated stimulations [3]. In early reports, increased c-MET expression was found in 87% of renal cell carcinomas and 70% of renal cancer samples from patients [4, 5]. c-MET amplification could be used as a prognostic marker in ovarian cancer patients with clear-cell adenocarcinoma [6]. EGFR overexpression has been associated with cancer cell proliferation, motility, migration, and invasion in various types of tumors, including breast, lung, colon, ovarian, and brain tumors [7]. Therefore, in clinical settings, EGFR inhibitors have been efficacious for the treatment of tumors associated with EGFR overexpression: lapatinib, an inhibitor of the kinase domain of EGFR, is used for metastatic breast cancers in conjunction with conventional cytotoxic drugs [8, 9].

In addition to cancer development and progression, RTK overexpression has been associated with anticancer drug resistance of tumors. HGF treatment in lung cancer cells induced cisplatin resistance via c-MET activation [10]. In non-adherent ovarian cancer cells, c-MET overexpression caused acquired resistance to cisplatin and paclitaxel through PI3K/AKT and extracellular signal-regulated kinase (ERK) 1/2 signaling [11]. In anticancer drug resistant small-cell lung cancer cells, c-MET expression was high and treatment with a c-MET-specific inhibitor SU11274 (SU112) sensitized cancer cells to cisplatin and paclitaxel [12]. Notably, c-MET overexpression was relevant for anti-EGFR therapy. Lung cancer cells that were initially sensitive to the EGFR kinase inhibitor gefitinib developed resistance via the amplification of c-MET. In line with this, c-MET amplification was observed in 22% of lung cancer samples that had developed anti-EGFR therapy [13]. Tumor samples from patients with resistance to anti-EGFR therapy such as gefitinib and erlotinib retained *EGFR* gene mutations such as T790M or exon 20 insertion [14, 15].

The nuclear factor (erythroid-derived 2)-like 2 (NFE2L2/NRF2) is a member of the cap’n’collar family of basic leucine-zipper (CNC-bZIP) transcription factors and is involved in the expression and regulation of various target genes, including detoxifying enzymes, antioxidant proteins, and drug efflux transporters [16, 17]. NRF2 activity is primarily regulated by the Kelch-like ECH-associated protein 1 (KEAP1), which interacts with NRF2 in the cytoplasm and mediates its Cullin3-based E3 ligase-dependent degradation [18, 19]. The NRF2-dependent upregulation of multiple genes involved in redox homeostasis and cellular detoxication endows normal cells cytoprotection against oxidative and electrophilic stress conditions [20, 21]. However, in many cancer cells, NRF2 is often overexpressed and the consequent elevation in xenobiotic detoxifying enzymes and redox modulating proteins confers protection of cancer cells from anticancer drug treatment, apoptotic stimuli, and radiotherapy [22, 23]. Particularly, several drug efflux transporters are known to be under the control of NRF2: the expression of the multidrug resistance gene (MDR1), multidrug resistance-associated protein-1 (MRP1), and breast cancer resistance protein (BCRP) was upregulated in cancer cells with NRF2 overactivation, which led to chemoresistance [24–26].

BCRP, the ATP-binding cassette G2 (ABCG2), has been related to resistance to anticancer drugs such as doxorubicin, daunorubicin, mitoxantrone, and topotecan [27, 28]. The fluorescent dye Hoechst 33342 (H342) is a substrate of BCRP and therefore, cellular H342 levels are often used as a marker of BCRP activity [29]. Although the regulatory molecules for BCRP expression are not fully understood, several transcription factors have been involved in *BCRP* gene expression: peroxisome proliferator-activated receptor-γ (PPARγ), progesterone receptor, hypoxia-inducible factor-1ɑ (HIF-1ɑ), and NRF2 [25, 30]. In addition, it was shown that the PI3K/AKT signaling pathway affects cellular levels of BCRP. In *akt* knockout mice, the number of H342-negative cells was decreased and the introduction of *akt* in these mutant cells restored H342-negative cell numbers [31]. The treatment of AKT inhibitors in hepatoma cells decreased the level of plasma membrane BCRP [32]. Therefore, the activation of the upstream molecules of PI3K/AKT can elevate BCRP activity. EGFR activation enhanced the level of plasma membrane BCRP in head and neck squamous cancer cells [33], and accordantly, treatment with EGFR inhibitor erlotinib reversed tumor resistance to topotecan by reducing the expression of BCRP/ABCG2 [34]. The association of c-MET with BCRP expression has been demonstrated by our recent report [35]. In doxorubicin-resistant ovarian carcinoma cells, BCRP overexpression was mediated by the c-MET elevation and resultant PI3K/AKT activation, and thus, the inhibition of c-MET could repress the plasma membrane BCRP level and enhanced doxorubicin-induced cell death. These indicate that BCRP elevation is one of molecular mechanisms of c-MET/EGFR-induced cancer resistance.

Recently, increasing attention is being given to molecular links between NRF2 and cancer cell signaling for cancer resistance. Particularly, several reports demonstrated the potential association of NRF2 with RTK signaling. EGFR ligand treatment induced NRF2 activation through the PI3K/AKT pathway in pulmonary alveolar cells [36]. In non-small-cell lung cancer (NSCLC), treatment with EGF or aberrant EGFR activation was shown to elevate NRF2 and its target gene expressions [37]. The HGF/c-MET signaling induced Nrf2-mediated gene expression in mice hepatocytes, which is involved in NADPH oxidase regulation, and the deletion of the c-MET gene disturbed cellular redox homeostasis [38]. These results raise an intriguing question of whether NRF2 signaling is correlated with c-MET and EGFR expression and consequent BCRP levels, ultimately modulating chemoresistance. To elucidate this, we examined the expression of c-MET and EGFR levels in *NRF2*-silenced cancer cells. In addition, we identified miRNA signature changes in *NRF2-*silenced cancer cells to investigate its relevance to BCRP modulation.

## RESULTS

### NRF2 inhibition decreases c-MET and EGFR levels in SKOV3 and A498

**Figure 1:**
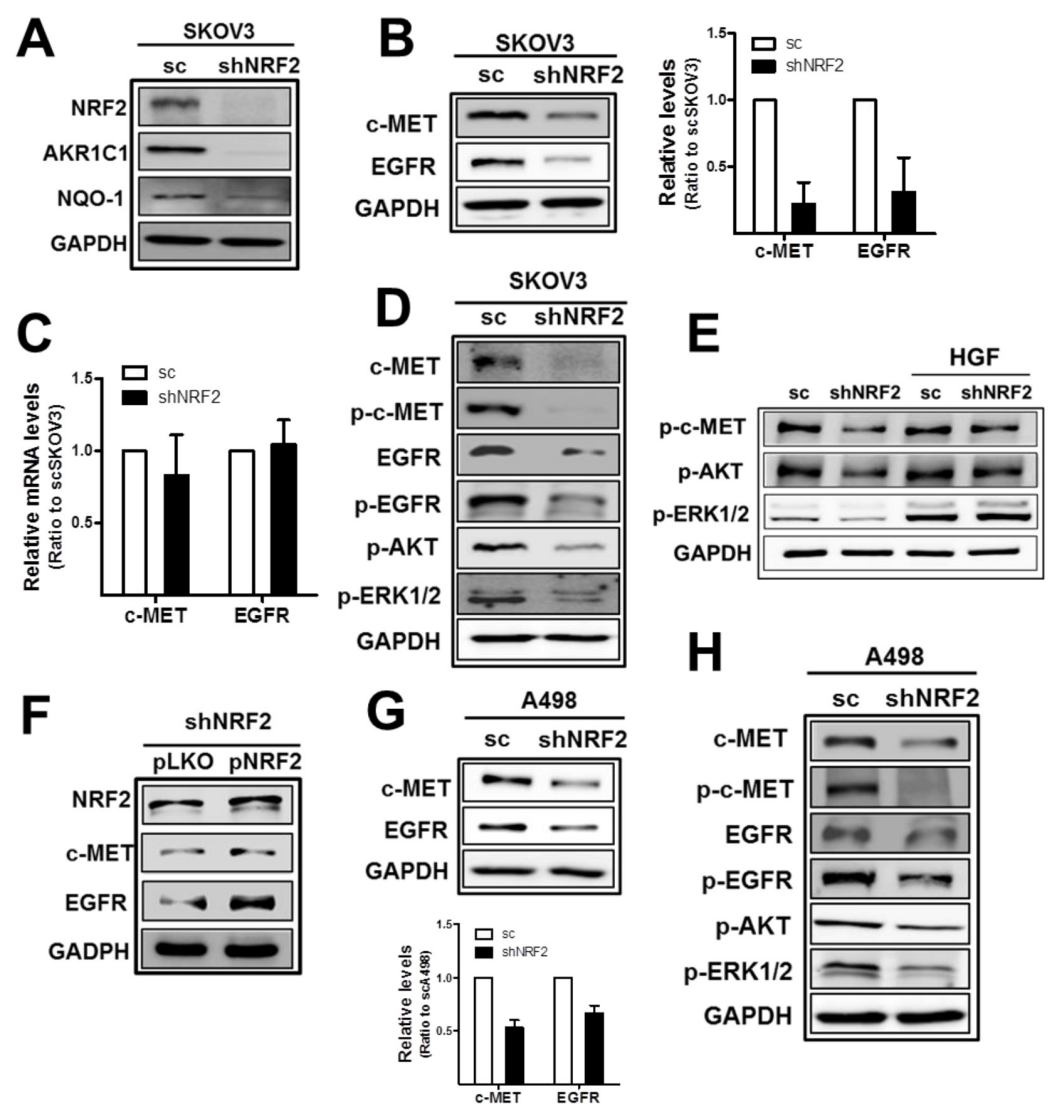
*NRF2*-silencing represses c-MET and EGFR levels in SKOV3 and A498 **(A)** The scSKOV3 and shNRF2-SKOV3 cell lines were established following stable transduction of cells with lentiviral particles containing either a nonspecific scRNA- or *NRF2* shRNA-expressing plasmid. NRF2-silencing was verified by measuring protein levels for NRF2, AKR1C1, and NQO-1. **(B)** c-MET, EGFR, and GAPDH protein levels in sc and shNRF2-SKOV3 cells were examined by Western blotting. The quantified relative levels are means ± standard deviation (SD) from three independent experiments. **(C)** Transcript levels for *c-MET* and *EGFR* were determined by relative real-time PCR. **(D)** The sc and shNRF2-SKOV3 cells were incubated with serum-free media (SFM) for 24 h and protein levels for c-MET, p-c-MET (Tyr1234/1235), EGFR, p-EGFR (Tyr1068), p-AKT (Ser473), and p-ERK1/2 (Thr202/Tyr204) were determined by Western blotting. **(E)** HGF (10 ng/ml) was incubated for 24 h in scSKOV3 and shNRF2-SKOV3 cells, and levels for p-c-MET, p-AKT, and p-ERK1/2 were determined. **(F)** Lentiviral particles with pLKO-NRF2 plasmid were introduced into shNRF2-SKOV3 cells for forced expression of NRF2. Levels for NRF2, c-MET and EGFR were assessed in the control (pLKO) and NRF2 rescue group (pNRF2). **(G)** c-MET, EGFR and GAPDH protein levels in sc and shNRF2-A498 cells were examined. Values are means ± SD from three independent experiments. **(H)** Protein levels for c-MET, p-c-MET, EGFR, p-EGFR, p-AKT, and p-ERK1/2 were monitored under serum-free media (SFM) condition. Similar blots were obtained from three independent experiments (A, D, E, F, and H).

In order to choose cancer cell lines that expressed high levels of both c-MET and EGFR, Western blot and real time polymerase chain reaction (PCR) analyses were performed using four cell lines: ovarian carcinomas SKOV3 and A2780, renal carcinoma A498, and breast carcinoma MCF7. Among these, SKOV3 and A498 were chosen because these cell lines expressed high levels of both c-MET and EGFR when compared to the other cell lines (Supplementary Figure 1A and 1B). Then, we investigated potential involvement of NRF2 in c-MET and EGFR regulation using *NRF2*-silenced SKOV3 and A498 cell lines. The stable SKOV3 cell line that expressed *NRF2*-specific shRNA (shNRF2-SKOV3) showed significantly lower levels of NRF2 and its target aldo-keto reductase 1C1 (AKR1C1) and NAD(P)H: quinone oxidoreductase-1 (NQO1) than the cell line that expressed the nonspecific scRNA expressing SKOV3 (scSKOV3), as indicated in Figure 1A and Supplementary Figure 2. Notably, in *NRF2*-silenced SKOV3, protein levels of c-MET and EGFR were substantially lower than those in scSKOV3 control cells (Figure 1B). Levels of c-MET and EGFR were decreased by 0.78- and 0.69-fold by *NRF2* knockdown in SKOV3. However, mRNA levels for c-MET/EGFR did not show a noticeable difference between scSKOV3 and shNRF2-SKOV3 (Figure 1C). In line with repressed total protein levels, phosphorylated c-MET (p-c-MET) and p-EGFR levels were significantly reduced in the serum-free medium-cultured *NRF2*-silenced SKOV3. Levels of p-AKT and p-ERK1/2 were also repressed in the shNRF2-SKOV3 (Figure 1D), which indicates lowered c-MET/EGFR downstream signaling in *NRF2*-silenced cancer cells. Whereas, HGF-inducible levels of p-AKT and p-ERK1/2 did not show noticeable differences between scSKOV3 and shNRF2-SKOV3 (Figure 1E). The direct involvement of NRF2 in c-MET/EGFR alterations was confirmed in NRF2 rescue experiment. The forced expression of NRF2 in shNRF2-SKOV3 elevated c-MET and EGFR levels when compared to the control plasmid group (Figure 1F). Similarly to SKOV3 cells, *NRF2*-silenced renal carcinoma A498 (shNRF2-A498) retained reduced levels of c-MET, EGFR, p-c-MET, p-EGFR, p-AKT, and p-ERK1/2 when compared to those of the A498 control cells (Supplementary Figure 3 and Figure 1G-1H).

Altered gene expression signature also indicated the reduced c-MET/EGFR signaling in *NRF2*-silenced ovarian cancer cells. When genes whose expression decreased more than 50% were analyzed using the KEGG database, 15 genes (*FGF18, FN1, ITGA1, ITGA2, ITGB4, ITGB6, ITGB8, KDR, KITLG, LAMB1, LPAR1, LPAR3, SPP1, SYK, VEGFA*) were associated with PI3K/AKT signaling pathway and 11 genes (*CACNA2D, DUSP4, FGF18, IL1R1, MAP2K6, MAP3K1, MECOM, PTPRR, RPS6KA6, TAOK3, TGFBR2*) belonged to MAPK signaling pathway (Table 2).

**Table 2:** The signaling pathway was analyzed using the KEGG database for genes whose expression decreased more than 50% by *NRF2*-silencing in SKOV3 cells

Rank	KEGG number	Related pathway (number of genes)
1	hsa01100	Metabolic pathways (40)
2	hsa05200	Pathways in cancer (20)
3	hsa04510	Focal adhesion (15)
3	hsa04151	PI3K-AKT signaling pathway (15)
5	hsa04360	Axon guidance (14)
6	hsa04810	Regulation of actin cytoskeleton (13)
6	hsa04060	Cytokine-cytokine receptor interaction (13)
8	hsa04010	MAPK signaling pathway (11)
8	hsa04014	Ras signaling pathway (11)
10	hsa04514	Cell adhesion molecules (10)

The signaling pathways are indicated in order of the number of genes involved. The number of genes that are associated with each signaling pathway is shown in parentheses.

As confirmatory evidence of the NRF2 effect on c-MET/EGFR, additional *NRF2*-specific shRNA (sh #2) was transiently expressed in A498, and repressed c-MET/EGFR expression was also observed (Supplementary Figure 4).

These results indicate the shRNA-mediated stable inhibition of NRF2 significantly reduced c-MET and EGFR levels, and thereby affected their downstream signaling in SKOV3 and A498 cell lines.

### MiR-206 level is increased in *NRF2*-silenced cells and c-MET/EGFR level was reduced by miR-206

In an attempt to investigate underlying molecular mechanisms of c-MET/EGFR reduction in *NRF2*-silenced cancer cells, we hypothesized the potential involvement of miRNAs in the dual regulation of c-MET and EGFR. First, the analysis with the miRNA–mRNA targets database (TarBase v7.0; http://diana.imis.athena-innovation.gr) retrieved 15 miRNAs that are likely to target both c-MET and EGFR (Table 3). Second, when levels of these miRNAs were assessed in the control and *NRF2*-silenced cells, levels of miR-133b, miR-206, and miR-542-3p were commonly elevated by more than 50% by *NRF2* knockdown in both SKOV3 and A498 cells (Table 3).

**Table 3:** MiRNAs that were predicted to target both *c-MET* and *EGFR*

miRNAs	Ratios (sh/sc) in SKOV3	Ratios (sh/sc) in A498
miR-1	1.04	n.d.
miR-130a-3p	1.56	0.87
**miR-133b**	**1.48**	**1.54**
miR-155-5p	0.26	n.d.
**miR-206**	**1.78**	**2.10**
miR-218-5p	0.03	n.d.
miR-23a-3p	0.66	n.d.
miR-27a-3p	0.51	n.d.
miR-30a-5p	0.46	n.d.
miR-301b-3p	1.27	n.d.
miR-34a-5p	0.90	n.d.
miR-454-3p	0.88	n.d.
miR-522-5p	0.45	n.d.
**miR-542-3p**	**2.24**	**1.73**
miR-let-7a-5p	0.76	n.d.

Levels of miRNAs were determined using real time RT-PCR analysis and ratios of miRNA levels of shNRF2 to the levels of sc control were obtained (n.d., not determined).

The above results indicated that miR-133b, miR-206, and miR-542-3p are the *NRF2*-silencing-inducible miRNAs, and therefore, might be responsible for the dual inhibition of c-MET and EGFR in shNRF2 cancer cells. To investigate this, mimic or inhibitor nucleotides for each miRNA were transfected into scSKOV3 or shNRF2-SKOV3, and levels of c-MET and EGFR were monitored. Results showed that miR-133 and miR-542-3p did not target c-MET/EGFR in our system. The miR-133 mimic transfection into scSKOV3 did not reduce c-MET/EGFR levels, and miR-133 (miR-133-3p and miR-133-5p) inhibitor transfection into shNRF2-SKOV3 did not alter c-MET/EGFR levels (Supplementary Figure 5A). Similarly, miR-542-3p transfection did not affect the levels of c-MET and EGFR (Supplementary Figure 5B).

**Figure 2:**
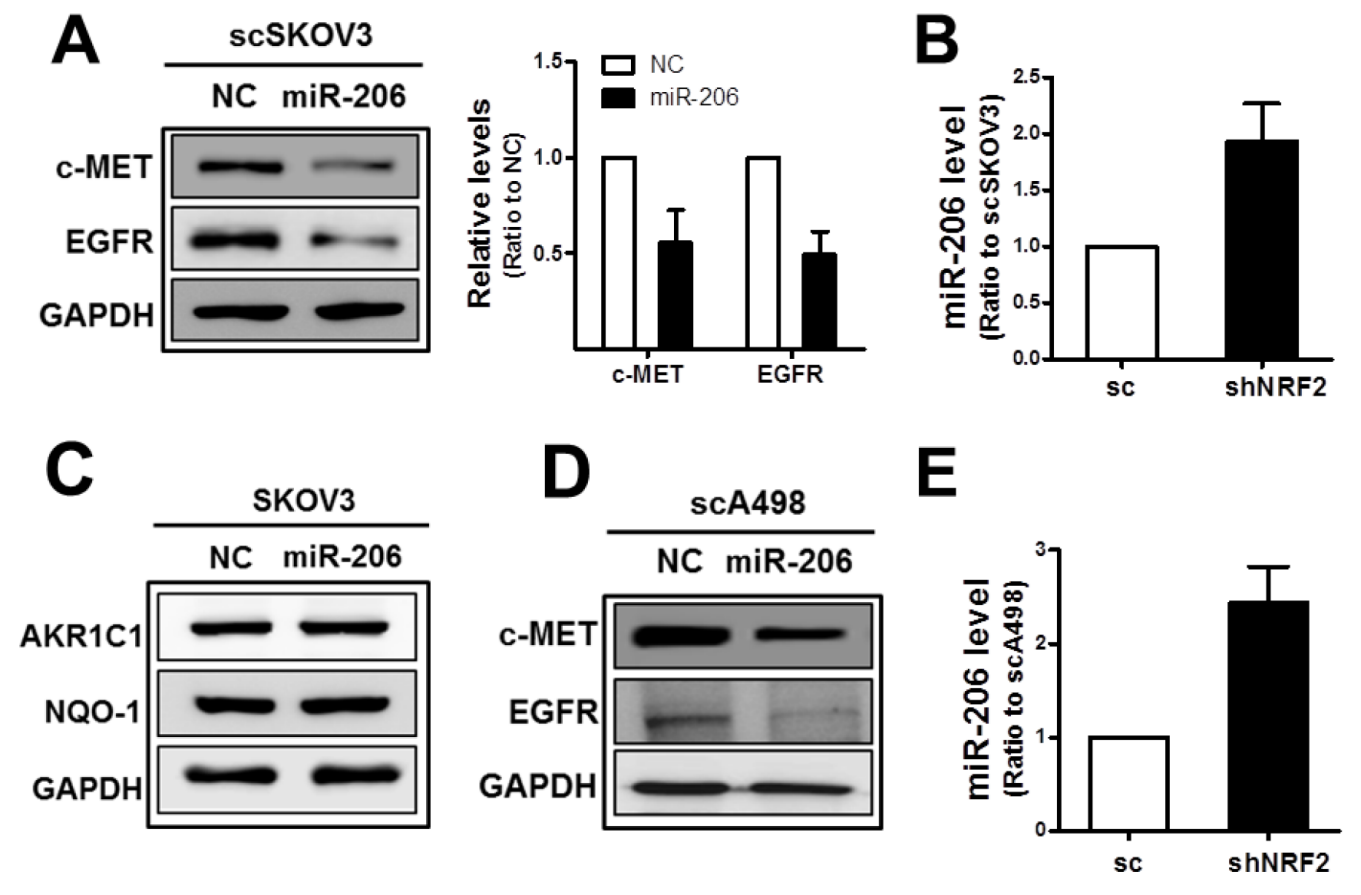
MiR-206 mimic treatment represses c-MET and EGFR levels **(A)** The scSKOV3 cells were transfected with miR-206 mimic (100 nM) or negative control (NC), and c-MET, EGFR and GAPDH protein levels were determined by Western blot. Quantified results are means ± SD from three independent experiments. **(B)** MiR-206 levels in the sc and shNRF2-SKOV3 were confirmed using relative real-time PCR analysis. Values are means ± SD from three experiments. **(C)** Protein levels for AKR1C1 and NQO-1 were assessed in miR-206 expressing SKOV3 cells. **(D)** c-MET, EGFR, and GAPDH protein levels were determined by Western blotting after transfection of scA498 cells with miR-206 mimic or negative control (NC). **(E)** MiR-206 levels in the sc and shNRF2-A498 cells were confirmed using relative real-time PCR analysis. Values are means ± SD from three experiments. Similar blots were obtained from three independent experiments (B, C, and D).

The subsequent experiment demonstrated that miR-206 was associated with dual control of c-MET and EGFR in our system. Transfection of the scSKOV3 with miR-206 mimics repressed protein levels of c-MET as well as of EGFR (Figure 2A), and higher miR-206 levels were confirmed in the shNRF2-SKOV3 cells when compared to those in the control cells (Figure 2B). In miR-206 transfected cells, levels of NRF2 target genes AKR1C1 and NQO1 were not changed (Figure 2C). Similar to SKOV3, protein levels of c-MET and EGFR were diminished and the miR-206 level was elevated in *NRF2*-silenced A498 cells when compared to that of the control cells (Figure 2D and 2E). These results suggest that miR-206, the *NRF2*-silencing-inducible miRNA, affects the levels of c-MET and EGFR.

### c-MET/EGFR reduction in *NRF2*-silenced cells is mediated by miR-206 elevation

**Figure 3:**
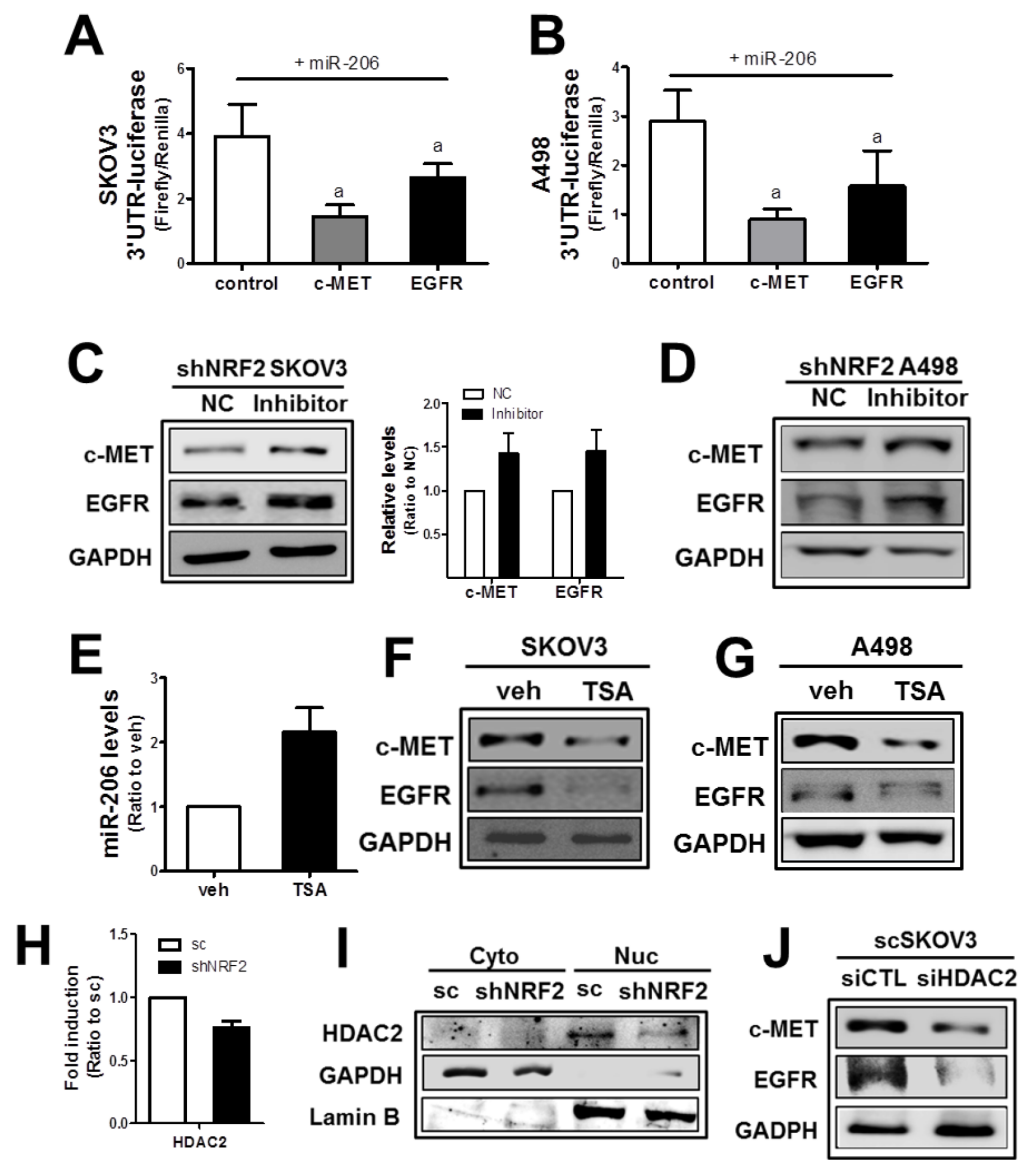
The *NRF2*-silencing-induced miR-206 directly mediates c-MET and EGFR reductions **(A-B)** SKOV3 (A) or A498 (B) cells were co-transfected with miR-206 and the *c-MET*-3′-UTR- or *EGFR*-3′UTR-luciferase reporter plasmid. After 18 h, 3-UTR-derived luciferase activity was measured. Values are means ± SD from 4 experiments. (C-D) After transfection of shNRF2-SKOV3 **(C)** or shNRF2-A498 **(D)** cells with the miR-206 inhibitor (100 nM) or negative control (NC), c-MET and EGFR protein levels were assessed by Western blotting. Data are means ± SD from three independent experiments. **(E)** The scSKOV3 cells were incubated with trichostatin (TSA, 0.3 μM) for 18 h, and miR-206 levels were measured using real-time PCR analysis. Values are means ± SD from three experiments. **(F-G)** Protein levels for c-MET and EGFR were determined in SKOV3 (I) and A498 (J) following the incubation with TSA (0.3 μM) for 24 h. **(H)** The transcript level of HDAC2 was assessed in sc and shNRF2-SKOV3 cells. **(I)** Cytoplasmic (Cyto) and nuclear (Nuc) levels of HDAC2 were determined Western blotting. **(J)** The scSKOV3 cells were transiently transfected with nonspecific siRNA (siCTL) or *HDAC2*-specific siRNA (siHDAC2) and the protein levels for c-MET and EGFR were assessed by Western blotting. Similar blots were obtained from three independent experiments (D, F, G, I and J).

The direct involvement of miR-206 in the expression of c-MET and EGFR was confirmed in the 3’-untranslated region (3′-UTR)-mediated luciferase analysis. In target analysis with TargetScan (http://www.targetscan.org/vert_71/) and Diana Tools (http://diana.imis.athena-innovation.gr/DianaTools/index.php?r=tarbase/index), miR-206 was predicted to bind to the 3′-UTR of the *c-MET* as well as the *EGFR* gene (Supplementary Figure 6). To test the specific regulation of the *c-MET* and *EGFR* genes through these predicted binding sites, the 2,300 bp-length 3′-UTR region of the *c-MET* and the 1,700 bp-length 3′-UTR region of the *EGFR* gene were cloned into the luciferase reporter plasmid. The 3′-UTR-luciferase plasmids were transfected into cancer cells to monitor luciferase activity under miR-206 overexpression. In SKOV3, luciferase activity from both the *c-MET* and *EGFR* 3′-UTR was significantly lowered by miR-206 mimic transfection when compared to that of the nonspecific miRNA control (Figure 3A), indicating a direct interaction between miR-206 and c-MET*/EGFR* 3′-UTR. Similarly, 3′-UTRs of the *c-MET* and *EGFR* were found to interact with miR-206 in A498 cells (Figure 3B).

Next, to confirm the association of miR-206 with c-MET/EGFR in *NRF2*-silenced cancer cells, shNRF2-SKOV3 and shNRF2-A498 were transfected with the miR-206 inhibitor nucleotides. Results showed that the miR-206 inhibitor treatment restored c-MET and EGFR levels in both cell lines (Figure 3C and 3D), which indicate direct involvement of miR-206 in repressed c-MET/EGFR expression in *NRF2*-silenced cells.

Next, in order to investigate underlying mechanisms of NRF2-dependent expression of miR-206, we speculated epigenetic regulation of miR-206. A recent report has demonstrated that histone deacetylase-4 (HDAC4) repressed miR-206 expression [39]. To test the involvement of HDAC in miR-206 regulation, SKOV3 and A498 cells were treated with a HDAC inhibitor trichostatin (TSA) and levels of miR-206 and c-MET/EGFR were assessed. Results indicated that TSA treatment clearly elevated the miR-206 level and reduced c-MET/EGFR levels (Figure 3E, 3F and 3G). Next, levels of HDAC1, 2, 4, and 6 were determined in scSKOV3 and shNRF2-SKOV3 cells to identify responsible HDAC subtype for miR-206 elevation. HDAC1 and HDAC6 levels were similar in both cell lines. HDAC4 level was higher in shNRF2-SKOV3, which is not in agreement with miR-206 elevation (Supplementary Figure 7A and 7B). Notably, transcript and protein levels of HDAC2 were lower in shNRF2 SKOV3 compared with those in scSKOV3 (Figure 3H and 3I). Moreover, silencing of *HDAC2* in SKOV3 repressed c-MET and EGFR levels. These results suggested the potential involvement of HDAC2 in miR-206 expression in *NRF2*-silenced SKOV3.

### MiR-206 overexpression exhibits an antitumor effect

**Figure 4:**
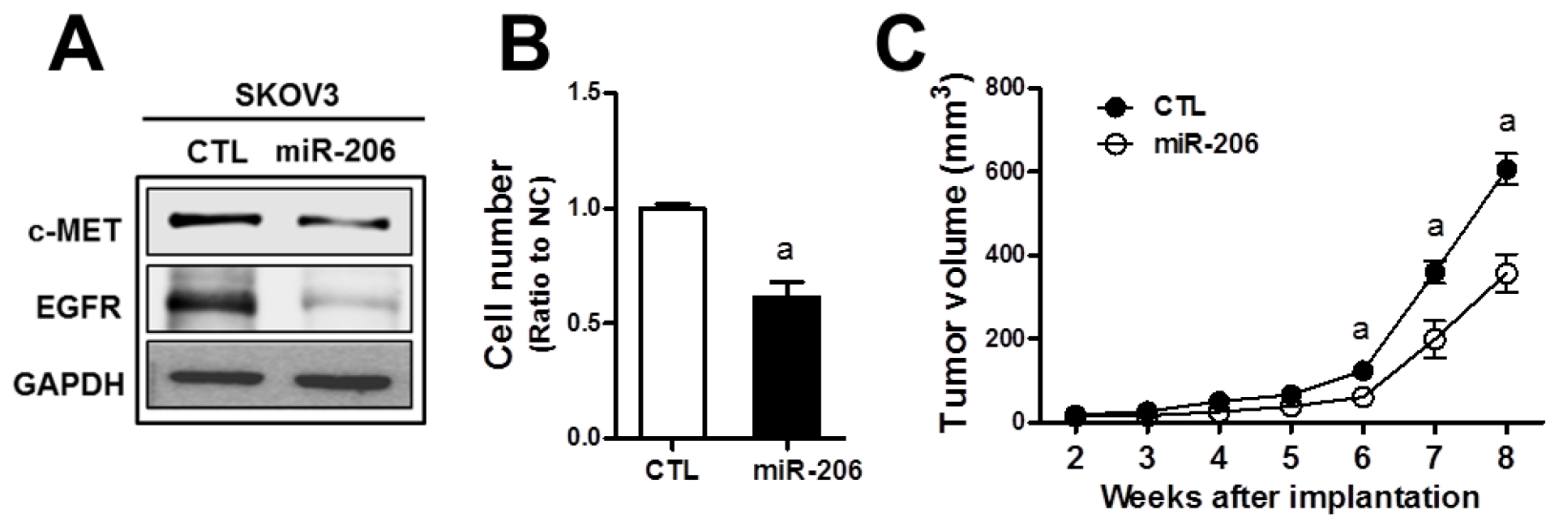
MiR-206 exhibits an antitumor effect in SKOV3 **(A)** SKOV3 cells were stably transduced with lentiviral particles containing miR-206- or negative control miRNA-expression plasmid. Levels of c-MET and EGFR protein were assessed by Western blotting in these two cell lines (SKOV3-CTRL and SKOV3-miR-206). **(B)** Proliferation rate of the SKOV3-CTRL and SKOV3-miR-206 cells was assessed using a TC10 Automated Cell Counter. Values are means ± SD from three experiments. **(C)** The SKOV3-CTRL and SKOV3-miR-206 were implanted in BALB/c-nu/nu mice and tumor growth was monitored weekly for 8 weeks. Tumor volume was calculated by the formula V = (a^2^ x b)/2 (a is the width and b is the length in mm). Each group contained four to five animals. Values are means ± SE. Similar blots were obtained from three independent experiments (A).

c-MET/EGFR signaling is a strong stimulator for cancer proliferation and survival, and therefore, the miR-206-mediated dual repression of c-MET and EGFR can confer antitumor effects. To test this, we established the stable SKOV3 cell line (SKOV3-miR206) that overexpressed miR-206 using the miR-206-containing lentivirus expression plasmid. In established stable cells, we could confirm reduced expressions of c-MET and EGFR (Figure 4A). In the measurement of cell proliferation, the SKOV3-miR206 cells showed a lower rate of cell growth than that of control miRNA-expressing cells (CTL) (Figure 4B). When the control and SKOV3-miR206 cells were inoculated in BALB/c-nu/nu mice, significant tumor growth retardation was observed in SKOV3-miR206 compared to that of the control cells (Figure 4C). These results show that miR-206 exerts antitumor effects in cancer cells.

**Figure 5:**
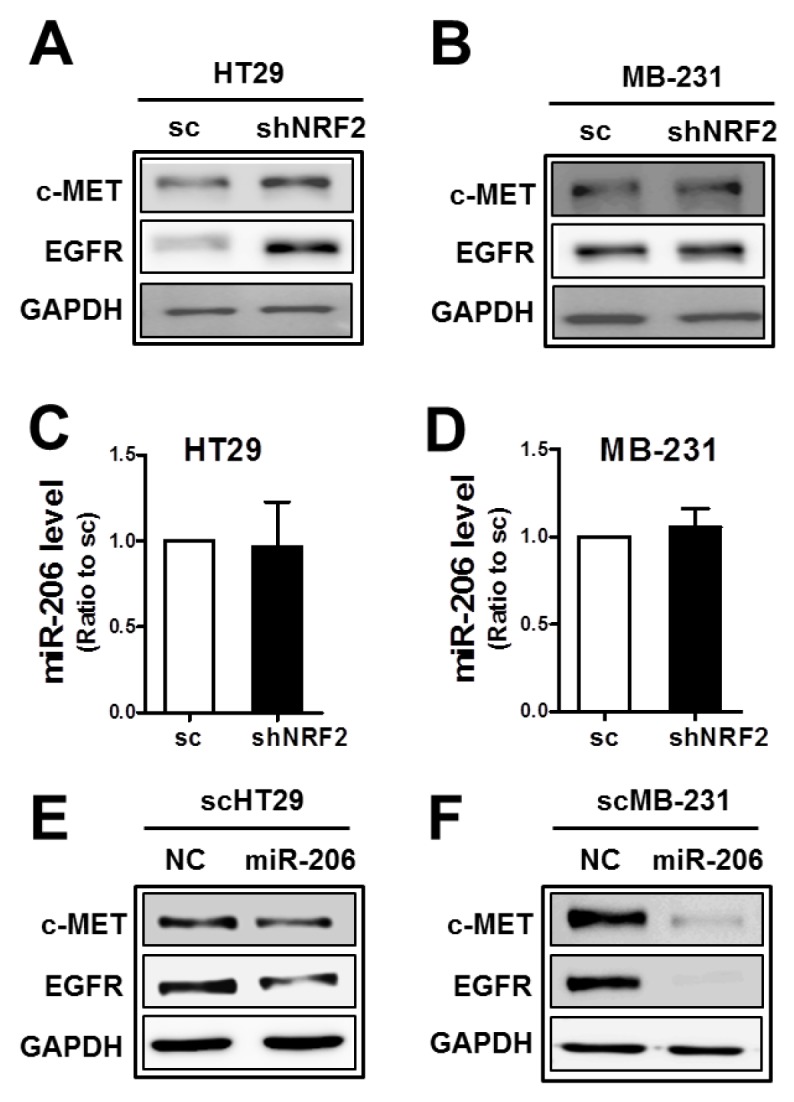
The association of NRF2 with miR-206 is cancer cell type-dependent **(A-B)** c-MET and EGFR protein levels were determined in *NRF2*-silenced HT29 (shNRF2-HT29; A) and *NRF2*-silenced MDA-MB231 (shNRF2-MB231; B) cells. **(C-D)** MiR-206 levels were assessed by real-time PCR analysis in shNRF2-HT29 (C) and shNRF2-MB-231 cells (D). Values are means ± SD from three experiments. **(E-F)** After transfection of the scHT29 (E) and scMB-231 (F) with the miR-206 mimic (100 nM), protein levels for c-MET and EGFR were assessed by Western blotting. Similar blots were obtained from three independent experiments (A, B, E, and F).

Since our results showed a dual inhibitory role of miR-206 in c-MET and EGFR expression in *NRF2*-silenced SKOV3 and A498 cells, we asked whether this was a common biological link in other types of cancer cells. Unlike SKOV3 and A498, levels of c-MET and EGFR were not lowered by *NRF2* knockdown in colorectal carcinoma HT29 and breast carcinoma MDA-MB231 cells (Figure 5A and 5B). Of note, miR-206 levels were not altered by *NRF2* silencing in these cells (Figure 5C and 5D), although the introduction of miR-206 into these cells suppressed c-MET and EGFR levels (Figure 5E and 5F). These results suggest that association of NRF2 activity with miR-206 upregulation is a cancer cell type-specific biological phenomenon.

### c-MET/EGFR reduction is associated with BCRP decrease

**Figure 6:**
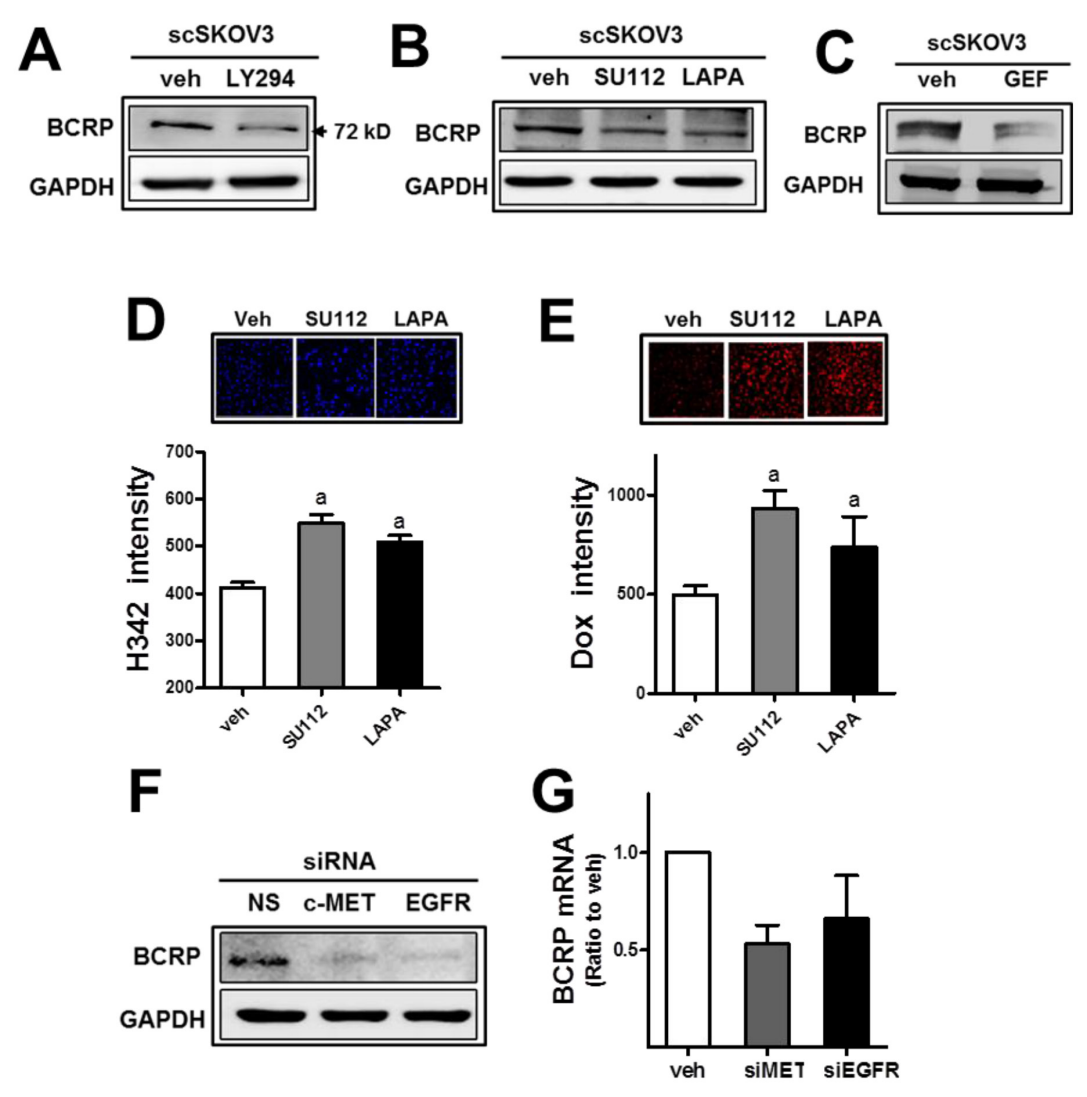
The inhibition of c-MET or EGFR leads to BCRP reduction and doxorubicin sensitization **(A)** The scSKOV3 cells were incubated with PI3K inhibitor (LY294; 5 μM) and protein levels for BCRP were assessed by Western blotting. **(B-C)** After incubation of the scSKOV3 with pharmacological inhibitor of c-MET (SU112; 1 μM) or EGFR (LAPA; 2 μM, GEF; 5 μM), BCRP protein levels were determined. **(D-E)** The scSKOV3 cells were incubated with SU112 or LAPA, and cellular accumulation levels of Hoechst 33342 (H342; 2 μg/ml for 30 min; D) or doxorubicin (Dox; 2 μM for 6 h; E) were monitored. Cellular fluorescent intensities were quantified using a Cell Insight system. Values are means ± SD from 4 experiments. **(F)** The scSKOV3 cells were transiently transfected with *c-MET*-specific siRNA (siMET) or *EGFR*-specific siRNA (siEGFR), and the protein level for BCRP was assessed by Western blotting. **(G)** Transcript levels of BCRP were monitored using real-time PCR analysis after transfection with *c-MET*-specific siRNA (siMET) or *EGFR*-specific siRNA (siEGFR). Values are means ± SD from three experiments. Similar blots were obtained from three independent experiments (A, B, C and F).

As an additional consequence of miR-206-mediated c-MET/EGFR inhibition, attenuation of anticancer drug resistance was hypothesized. Since PI3K-associated signaling was shown to control drug efflux transporter BCRP levels in cancer cells, miR-206-induced c-MET/EGFR inhibition might affect BCRP in our cell system. First, the expression level of BCRP in SKOV3 was repressed by treatment with a PI3K inhibitor LY294002 (LY294; Figure 6A), confirming the link between PI3K signaling and BCRP. Second, pharmacological inhibitors of c-MET (SU112) and EGFR (lapatinib) diminished BCRP levels (Figure 6B). Additionally, gefitinib (GEF), a specific inhibitor of EGFR, showed BCRP repression as well (Figure 6C). Cellular accumulation of H342 and doxorubicin was enhanced following treatment with these inhibitors in SKOV3 (Figure 6D and 6E). Similar to the results of the pharmacological inhibitor treatment, the siRNA-mediated inhibition of c-MET or EGFR suppressed the BCRP level in SKOV3 (Figure 6F). Transcript levels for BCRP were reduced by the inhibition of c-MET or EGFR (Figure 6G). These results imply a strong association of c-MET/EGFR/PI3K signaling with BCRP expression in SKOV3.

### MiR-206 is a novel modulator of BCRP in *NRF2*-silenced cancer cells

**Figure 7:**
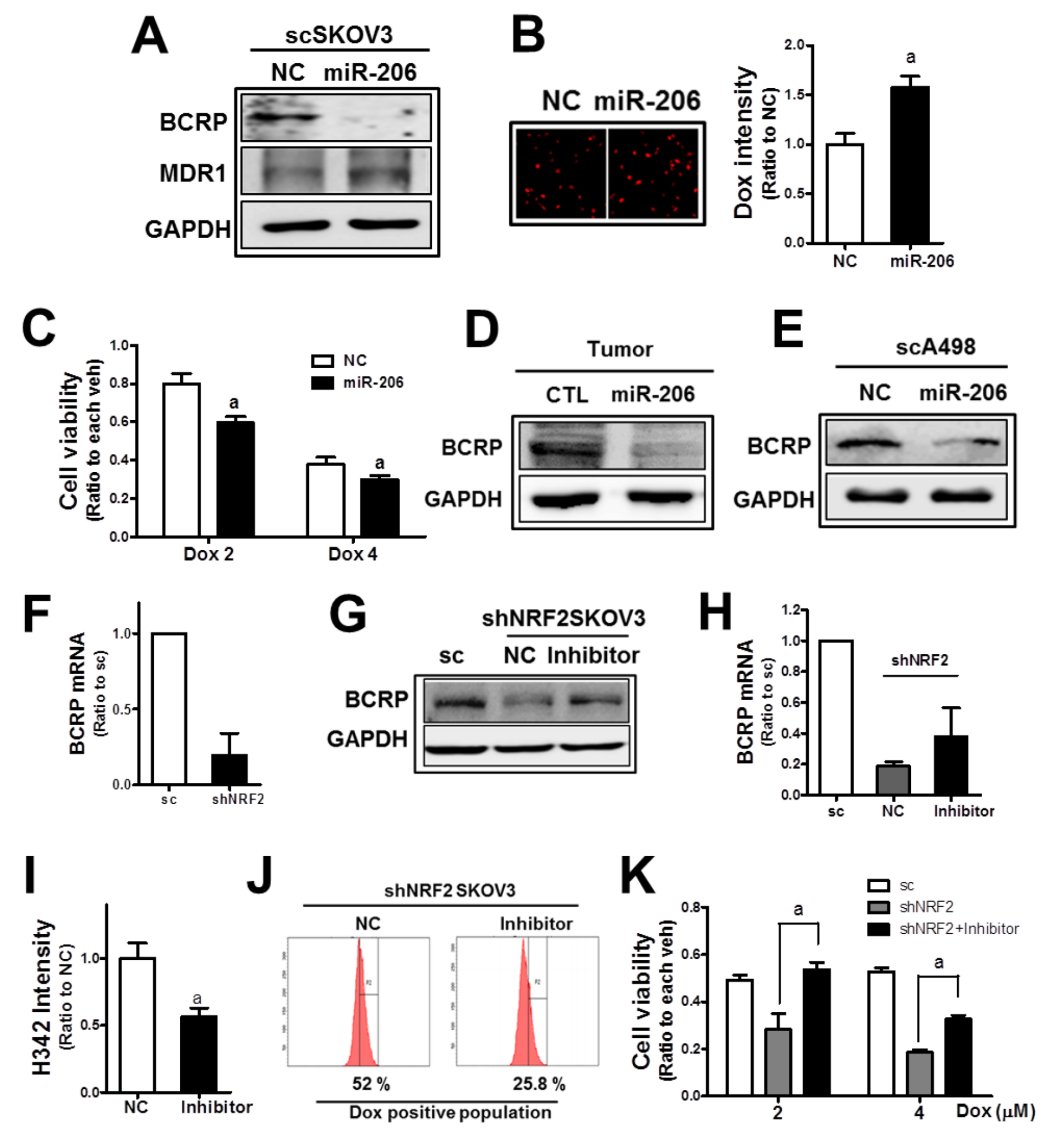
MiR-206 is a novel modulator of BCRP in *NRF2*-silenced cancer cells **(A)** Effects of the miR-206 mimic (100 nM) on BCRP and MDR1 were assessed by Western blotting. **(B)** The scSKOV3 cells were transfected with the miR-206 mimic and doxorubicin-derived fluorescent intensities were quantified using a Cell Insight system. Values are mean ± SD from 6 replicates. **(C)** MiR-206-transfected scSKOV3 cells were incubated doxorubicin (Dox; 2 and 4 μM) for 24 h, and cell viability was examined using MTT analysis. Values are means ± SD of 8 replicates. **(D)** Protein level of BCRP in tumors from the SKOV3-CTRL and SKOV3-miR206 cells. **(E)** BCRP protein level was assessed in scA498 after transfection with the miR-206 mimic. **(F)** BCRP mRNA levels were determined in sc and shNRF2-SKOV3 using relative quantification of RT-PCR. **(G)** The shNRF2-SKOV3 cells were transfected with the miR-206 inhibitor (100 nM), and BCRP protein levels were determined. **(H)** BCRP mRNA levels were assessed following miR-206 inhibitor (100 nM) transfection. **(I)** The shNRF2-SKOV3 cells were transfected with the miR-206 inhibitor and then incubated with H342 (2 μg/ml) for 30 min. After PBS washing, H342-derived fluorescent intensities were quantified. Values are means ± SD of 6 replicates. **(J)** The miR-206 inhibitor-treated shNRF2-SKOV3 cells were incubated with doxorubicin (Dox, 4 μM for 6 h), and cellular accumulation levels of doxorubicin were assessed using flow cytometric analysis. **(K)** Effect of the miR-206 inhibitor on the doxorubicin-induced cytotoxicity was evaluated. After transfection of shNRF2-SKOV3 cells with the miR-206 inhibitor, cells were incubated with doxorubicin (Dox, 2 and 4 μM). Viable cell numbers were determined after 24 h using MTT assay. Values are means ± SD from 8-10 replicates. Similar blots were obtained from three independent experiments (A, D, E, and F).

Next, the involvement of miR-206 in BCRP repression was examined. When miR-206 was transfected into SKOV3, the BCRP protein level was substantially repressed (Figure 7A). The level of another efflux transporter MDR1 was not repressed by miR-206. Reduced BCRP levels in miR-206-transfected SKOV3 led to a higher level of cellular doxorubicin accumulation and lower cell viability (Figure 7B and 7C). The association of miR-206 with BCRP expression was confirmed in tumor tissues. In tumors from the SKOV3-miR206, the BCRP level was lower than that in the control tumors (Figure 7D). In A498 cells, similar reductions by c-MET/EGFR inhibitor treatment on BCRP were observed, and the miR-206 mimic treatment repressed BCRP level (Figure 7E).

NRF2 is known to regulate BCRP expression through transcriptional regulation [25]. We also confirmed that mRNA level of BCRP was substantially lower in shNRF2-SKOV3 (Figure 7F). In addition to the transcriptional mechanism, miR-206-mediated BCRP repression may further contribute to BCRP regulation in *NRF2*-silenced SKOV3. In order to verify miR-206 contribution, the miR-206 inhibitor was transfected into *NRF2*-silenced SKOV3. The miR-206 inhibitor treatment increased BCRP levels, which is comparable to the control cell level (Figure 7G). Whereas, reduced transcript level of *BCRP* in knockdown cells was not fully recovered by miR-206 inhibitor treatment (Figure 7E), suggesting additional contribution of miR-206 to NRF2-mediated BCRP regulation. As a result of BCRP repression, cellular accumulation of H342 and doxorubicin was diminished in miR-206 inhibitor-treated cells (Figure 7I and 7J). Consequently, cell viability following doxorubicin treatment was significantly enhanced by the miR-206 inhibitor treatment in shNRF2-SKOV3 (Figure 7K). These results provide novel evidence of the role of miR-206 in BCRP inhibition and anticancer drug resistance, and clearly demonstrate the miR-206 contribution to BCRP reduction in *NRF2*-silenced cancer cells.

## DISCUSSION

In the current study, we demonstrated that the inhibition of NRF2 expression in ovarian and renal carcinoma cells led to the suppression of c-MET/EGFR levels through miR-206 upregulation, and the *NRF2*-silencing-induced miR-206 could reduce BCRP expression, resulting in chemosensitization to anticancer drug treatment. These findings provide insights into the molecular crosstalk between the cellular defense factor NRF2 and oncogene signaling pathway c-MET/EGFR, and further highlight the novel role of miR-206 in inhibiting BCRP expression.

The involvement of RTK signaling in BCRP expression has been demonstrated in multiple studies. Treatment of cancer cells with EGF could induce the mRNA and protein levels of BCRP through MAPK/extracellular regulated kinase 1/2 (ERK1/2) signaling [40]. PI3K/AKT signaling was found to be responsible for BCRP upregulation. In gefitinib-resistant cells, AKT-dependent phosphorylation of EGFR facilitated the translocation of EGFR to the nucleus, where it targets the promoter of the *BCRP* gene to enhance transcription of BCRP [41]. In line with these findings, several EGFR inhibitors were found to reverse tumor resistance by modulating BCRP. Erlotinib, which was approved for the treatment of non-small cell lung cancer (NSCLC) patients, reduced the expression of BCRP and affected intracellular topotecan accumulation [34]. The combined treatment of lapatinib with conventional chemotherapeutic agents such as cisplatin, topotecan, and doxorubicin showed synergistic effects on the inhibition of cancer cell growth by directly inhibiting BCRP-mediated drug efflux activity [42, 43]. In our previous report, c-MET expression was enhanced in the doxorubicin-resistant ovarian cancer cells, which affected BCRP levels via PI3K/AKT activation, suggesting a novel link between c-MET and BCRP-mediated resistance [35].

Our results showed that miR-206 plays a dual inhibitory role in c-MET and EGFR expression. The direct inhibitory effect of miR-206 on 3′-UTRs of *c-MET* and *EGFR* genes has been demonstrated (Figure 3A and 3B). Transfection of miR-206 repressed c-MET/EGFR levels in ovarian, renal, colorectal, and breast carcinoma cells (Figures 2A, 2D, 5E, and 5F). As a consequence of c-MET/EGFR inhibition, stable SKOV3 cells that expressed miR-206 displayed a retarded cell proliferation and tumor growth (Figure 4B and 4C). These results are in good agreement with the antitumor effects of miR-206 in previous reports. Since the first finding of miR-206 as a skeletal muscle specific myogenic miRNA, miR-206 expression has been observed to be decreased in various cancers, and its reduced level has been associated with enhanced tumor proliferation, migration, and metastasis [44–47]. As part of other underlying molecular mechanisms, miR-206 has been shown to target *KRAS*, annexin a2 (*ANAX2*), *NOTCH3*, cyclin D1, and c-MET [47–49]. In addition to antitumor effects, we provide novel evidence that miR-206 can inhibit tumor resistance by suppressing the c-MET/EGFR-BCRP axis. MiR-206 transfection diminished BCRP levels with concomitant increases in cellular doxorubicin accumulation and cytotoxicity (Figure 7A, 7B, and 7C). The link between miR-206 and chemoresistance can be supported by a recent report [50]. In cisplatin-resistant lung cancer cells, forced expression of miR-206 sensitized cells to cisplatin and reduced levels of MDR1.

One noticeable observation of this study is the association of NRF2 activity with oncogene signaling of c-MET and EGFR. The relationship between NRF2 and oncogene signaling has not been fully understood yet; however, several studies have focused on oncogene-induced NRF2 activation. For instance, mutations in the oncogenic alleles of *KRAS* (G12D), *B-RAF* (V619E) and *MYC* enhanced NRF2 transcription and subsequently lowered intracellular reactive oxygen species (ROS) level [51]. In human NSCLC cells, oncogenic activation of *KRAS* transcriptionally promoted NRF2 expression through the 12-O-tetradecanoylphorbol-13-acetate (TPA)-response element, demonstrating the involvement of the KRAS-NRF2 axis in cisplatin resistance [52]. In NSCLC cells with both wild-type *KEAP1* and *EGFR*, EGF treatment led to a dose-dependent activation of NRF2 through the phosphorylation of ERK and AKT [37]. Similarly, the inhibition of tyrosine kinase activity of EGFR suppressed NRF2 nuclear accumulation and ARE-mediated transcription via attenuation of the phosphorylation of AKT and ERK1/2 [53]. These reports highlight the role of oncogenes in NRF2 signaling, which can eventually contribute to oncogene-induced cancer resistance. In addition to these findings, our study has identified that NRF2 activity can affect oncogene signaling such as c-MET and EGFR through miR-206. Notably, in our analysis with cBioPortal for cancer genomics (http://www.cbioportal.org/), genetic alterations in both *NRF2* and *c-MET* were found in 39 (13%) of 311 patients with ovarian carcinoma and the co-occurrence was statistically significant (*p* = 0.03), which further supports the association of NRF2 with c-MET oncogene.

The potential link between redox state-related factors and miR-206 has been suggested by several reports. It was clarified that heme oxygenase-1 (HO-1) suppressed miR-206 levels in rhabdomyosarcoma cells and therefore, treatment with HO-1 inhibitor protoporphyrin IX inhibited cancer cell growth and vascularization through miR-206 elevation [39]. It was reported that activation of NRF2 signaling in cancer cells attenuated miR-206 expression, causing modulation in metabolism-related genes, including the pentose phosphate pathway and the tricarboxylic acid pathway [54]. As an underlying molecular mechanism of NRF2-mediated miR-206 regulation, our results suggested that HDAC2 might be responsible for miR-206 elevation. In SKOV3 and A498, HDAC inhibitor treatment increased miR-206 expression along with the decreases in c-MET and EGFR. We further showed that *NRF2* knockdown altered HDAC levels: HDAC2 level was significantly decreased in shNRF2-SKOV3 cells. We also demonstrated that *HDAC2*-silencing led to reductions in c-MET and EGFR. Similarly to our results, *NRF2* loss in lung cancer cells decreased HDAC4 and subsequently elevated miR-206 [54]. In line with these results, our study demonstrated that *NRF2*-silencing elevated miR-206 expression in SKOV3 and A498 cells, and HDAC2 was associated with miR-206 expression.

Altogether, our results indicate that the *NRF2* silencing-mediated miR-206 regulation could suppress BCRP levels through c-MET/EGFR modulation, providing an additional underlying molecular mechanism for the tumor sensitization by *NRF2* inhibition.

## METHODS

### Reagents

Doxorubicin hydrochloride, LY294002 (LY294; 2-morpholin-4-yl-8-phenylchromen-4-one), SU11274 (SU112; (3Z)-N-(3-chlorophenyl)-3-(3,5-dimethyl-4-[(4-methylpiperazin-1-yl)carbonyl]-1H-pyrrol-2-ylmethylene)-N-methyl-2-oxo-2,3-dihydro-1H-indole-5-sulfonamide), 3-(4,5-dimethylthiazol-2-yl)-2,5-diphenyltetrazolium bromide (MTT), and puromycin were purchased from Sigma-Aldrich (Saint Louis, MO). Hoechst 33342 (H342; 2’-[4-ethoxyphenyl]-5-[4-methyl-1-piperazinyl]-2,5’-bi-1H-benzimidazole trihydrochloride trihydrate) was purchased from Life Technologies (Carlsbad, CA). The SYBR Premix ExTaq system was obtained from Takara (Otsu, Japan). Antibodies recognizing c-MET, p-c-MET (Tyr1234/1235), EGFR, p-EGFR (Tyr1068), PI3K (PI3K regulatory subunit-1a), AKT, p-AKT (Ser-473), ERK1/2, p-ERK1/2 (Thr202/Tyr204) and BCRP/ABCG2 were obtained from Cell Signaling Technology (Danvers, MA). Antibodies for NQO-1, NRF2, HDAC1, HDAC2, HDAC4, and glyceraldehyde-3-phosphate dehydrogenase (GAPDH) were purchased from Santa Cruz Biotechnology (Santa Cruz, CA). AKR1C1 antibody was from Abnova (Taipei City, Taiwan). Lapatinib ditosylate (N-[3-chloro-4[(3-fluorophenyl)methoxy-phenyl]-6-[5-[(2-methylsulfonylethylamino)methyl]furan-2-yl]quinazolin-4-amine ditosylate) and Gefitinib was obtained from Santa Cruz Biotechnology and Tocris Bioscience (Ellisville, MO, USA), respectively.

### Cell culture

Human ovarian carcinoma cell line SKOV3 was obtained from the Korean Cell Line Bank (Kwanak-gu, Seoul, Republic of Korea) and maintained in RPMI 1640 (GE Healthcare Life Sciences, Logan, UT) with 10% fetal bovine serum (FBS; GE Healthcare Life Sciences) and penicillin/streptomycin (WelGene Inc., Daegu, Republic of Korea). The renal carcinoma cell line A498, and breast carcinoma MCF7 and MDA-MB231, colon carcinoma HT29 were obtained from the American Type Culture Collection (Rockville, MD), and were cultured in Dulbecco’s modified Eagle’s medium (DMEM; GE Healthcare Life Sciences) or RPMI 1640. The A2780 cell line was purchased from the European Collection of Cell Culture (Salisbury, Wiltshire, UK) and maintained in RPMI 1640. All cells were grown at 37 °C in a humidified 5 % CO_2_ atmosphere.

### Stable or transient silencing of *NRF2*

*NRF2*-knockdown cancer cell lines were established as previously reported [55, 56]. Briefly, cells were incubated with lentiviral particles containing *NRF2*-specific shRNA construct 1 (designated as shNRF2; 5′- CCGGGCTCCTACTGTGATGTGAAATCTCGAGATTTCACATCACAGTAGGAGCTTTTT-3′) and subsequently placed under puromycin selection for 4 weeks. The corresponding scrambled RNA (scRNA)-transfected control cell lines were scSKOV3, scA498, scMDA-MB-231, and scHT29 cells, respectively. For the transient silencing, A498 cells were incubated with cholesterol for 15 min and the lentiviral particle containing *NRF2*-specific shRNA #2 (designated as shNRF2-#2; 5′- CCGGCCGGCATTTCACTAAACACAACTCGAGTTGTGTTTAGTGAAATGCCGGTTTTT-3′) was added to each well. After 48 h incubation, viral particle-containing media were removed and cells were recovered in the fresh medium for overnight.

### Pathway analysis of differential gene expression signature between sc and shNRF2-SKOV3

The global gene expression signatures were obtained from the sc control and *NRF2*–silenced SKOV3 as described previously [56]. The signaling pathway was analyzed using the Kyoto Encyclopedia of Genes and Genomes (KEGG) database (www.genome.jp/kegg/) for genes whose expression decreased more than 50% by *NRF2*-silencing.

### Stable miR-206 expression

SKOV3 stable cell line that expresses miR-206 (SKOV3-miR206) was established using the PLKO-miR-206 lentiviral construct (Sigma-Aldrich, Saint Louis, MO) and puromycin-selection.

### Transfection with the miRNAs mimic, inhibitor, or siRNAs

The AccuOligo human miR-206 mimic/inhibitor, miR-133b mimic/inhibitor, miR-542 mimic, and each negative control were obtained from the Bioneer Corporation. Predesigned siRNAs for *c-MET* (5’-GUGAAGAUCCCAUUGUCUA-3’ and 5’-UAGACAAUGGGAUCUUCAC-3’), *EGFR* (5’-GCAGAGGAAUUAUGAUCUU-3’ and 5’-AAGAUCAUAAUUCCUCUGC-3’), *HDAC2* (5’-GACGGAAACUGAGCUCAGU-3’ and 5’-ACUGAGCUCAGUUUCCGUC-3’), and a scrambled control siRNA were purchased from the Bioneer Corporation. For transfection, cells in 60 mm plates were transfected with the miRNAs mimic, miRNAs inhibitor or siRNAs using a Lipofectamine 2000 reagent (Life Technologies, Carlsbad, CA) as described previously [57].

### Total RNA isolation and real-time reverse transcriptase (RT)-PCR

Total RNAs were isolated using the TRIzol reagent (Thermo Fisher Scientific Inc., Waltham, MA) and used for the synthesis of cDNA as described previously [58]. For relative quantification of the mRNA levels, PCR analyses were performed with a LC480 Roche LightCycler (Roche Diagnostics Deutschland GmbH, Mannheim, Germany) with a Takara SYBR Premix ExTaq system (Takara Bio Inc., Kusatsu, Japan). Primers were synthesized by Bioneer (Daejeon, Republic of Korea) and the primer sequences for the human genes are: *NRF2*, 5′-ATAGCTGAGCCCAGTATC-3′ and 5′-CATGCACGTGAGTGCTCT-3′; *AKR1C1*, 5′-CGAGAAGAACCATGGGTGGA-3′ and 5′-GGCCACAAA-GGACTGGGTCC-3′; *NQO1*, 5′- CAGTGGTTTGGAGTCCCTGCC-3′ and 5′-TCCCCGTGGATCCCTTGCAG-3′; *c-MET*, 5’-TATGTGGCTGGGACTTTGGA-3’ and 5’-GCTTATTCATGGCAGGACCAAC-3’; *EGFR*, 5’- CGAATGGGCCTAAGATCCCG -3’ and 5’-AGCTTGGTTGGGAGCTTCTC-3’; *BCRP/ABCG2*, 5’-CACAACCATTGCATCTTGGCTG-3’ and 5’-TGAGAGATCGATGCCCTGCTTT-3’; *HDAC1*, 5′-TGCTAAAGTATCACCAGAGGGT-3′ and 5′-TGGCCTCATAGGACTCGTCA-3′; *HDAC2*, 5′-ATGGCGTACAGTCAAGGAGG-3′ and 5′-TCATTTCTTCGGCAGTGGCT-3′; *HDAC4*, 5′-CCCAGCACGGTGGATGTG-3′ and 5′-GGATCTGCCTCTGGATCTGC-3′; hypoxanthine phosphoribosyltransferase-1 (*HPRT1*), 5′-TGGCGTCGTGATTAGTGATG-3′ and 5′-GCTACAATGTG-ATGGCCTCC-3′.

### MicroRNA (miRNA) isolation and real-time RT-PCR

Small RNA-rich samples were isolated from cells with the TRIzol reagent or mirVana miRNA Isolation kit (Thermo Fisher Scientific Inc.) according to the manufacturer’s protocol. cDNAs were synthesized with a miScript RT kit (Qiagen GmbH, Hilden, Germany), and PCR was performed with a LightCycler using miScript SYBR green PCR kit (Qiagen GmbH) as described previously [35, 55]. Forward sequences for the miRNAs primer are described in Table 1.

**Table 1:** Forward primer sequences for the real-time PCR analysis of miRNAs levels

miRNAs	Forward primers
miR-1	5′-UGGAAUGUAAAGAAGUAUGUAU-3′
miR-130a-3p	5′-CAGUGCAAUGUUAAAAGGGCAU-3′
miR-133b	5′-UUUGGUCCCCUUCAACCAGCUA-3′
miR-155-5p	5′-UUAAUGCUAAUCGUGAUAGGGGU-3′
miR-206	5′-UGGAAUGUAAGGAAGUGUGUGG-3′
miR-218-5p	5′-UUGUGCUUGAUCUAACCAUGU-3′
miR-23a-3p	5′-AUCACAUUGCCAGGGAUUUCC-3′
miR-27a-3p	5′-UUCACAGUGGCUAAGUUCCGC-3′
miR-30a-5p	5′-UGUAAACAUCCUCGACUGGAAG-3′
miR-301b-3p	5′-CAGUGCAAUGAUAUUGUCAAAGC-3′
miR-34a-5p	5′-UGGCAGUGUCUUAGCUGGUUGU-3′
miR-454-3p	5′-UAGUGCAAUAUUGCUUAUAGGGU-3′
miR-522-5p	5′-CUCUAGAGGGAAGCGCUUUCUG-3′
miR-542-3p	5′-UGUGACAGAUUGAUAACUGAAA-3′
miR-let-7a-5p	5′-UGAGGUAGUAGGUUGUAUAGUU-3′

### Viable cell count

To determine cytotoxicity, cells were incubated with varied concentrations of doxorubicin and then MTT solution (2 mg/ml) was added for a further 4 h-incubation. Cell viability was monitored by measuring absorbance at 540 nm using a Spectro-star Nano microplate reader (BMG Lab Technologies, Offenburg, Germany) with the addition of 100 μl of dimethyl sulfoxide. Additionally, viable cell number was counted using a TC10 Automated Cell Counter (Bio-Rad Laboratories, Inc., Hercules, CA) in the presence of the trypan blue dye.

### Preparation of total cell lysates and cytoplasmic/nuclear extracts

Cells were treated with vehicle or drugs for indicated time periods and lysed with RIPA buffer (1 M, pH 7.4, Tris, 2M NaCl, 1M EDTA, 10% NP40 and protease/phosphatase inhibitors cocktail) by freeze-thawing cycles. Total cell lysates were prepared following centrifugation at 10,000g for 15 min at 4 °C. Protein concentration was determined by Pierce BCP protein assay kit (Thermo Scientific, Rockford, IL, USA). Nuclear and cytoplasmic extracts were prepared using NE-PER nuclear and cytoplasmic extraction reagents (Thermo Scientific) according to the manufacturer’s instructions.

### Western blot analysis

Proteins were run on 6–12% SDS–polyacrylamide gels and transferred to nitrocellulose membranes (Whatman, Dassel, Germany). Membranes were blocked with 3% bovine serum albumin (BSA) for 1 h and then, incubated with the corresponding primary and secondary antibodies. The chemiluminescence images from the antibody reaction were captured using a LAS-4000 Mini (Fujifilm, Tokyo, Japan).

### Measurement of the 3’-UTR-derived luciferase activity

The 2,300 bp 3’-UTR of the human *c-MET* or 1,700 bp 3’-UTR of the human *EGFR* was cloned into the pMirTarget vector (Origene, Rockville, MD) to produce the 3-UTR-luciferase plasmids. The 3’-UTR-*c-MET*, 3’UTR-*EGFR* or the empty pMirTarget vector was co-transfected with miR-206 mimic (100 nM) in SKOV3 and A498 cells, and luciferase activities were measured in cell lysates using the Dual Luciferase Assay System (Promega Corporation, Madison, WI) with a 20/20^n^ luminometer (Turner Designs, Sunnyvale, CA) [57].

### Measurement of intracellular accumulation of doxorubicin and H342

Cells in a 96-well flat bottom plate were incubated with doxorubicin for 6 hrs and washed with phosphate buffered saline (PBS). After the nuclear staining with H342 (2 μg/ml) for 30 min, the fluorescent intensities from doxorubicin (548/680 nm) and H342 (350/461 nm) were quantified using a Cell Insight Personal Cell Imager (Thermo Fisher Scientific Inc.) and the corresponding software as described previously [35]. Cellular accumulation of doxorubicin was also determined using a flow cytometry (Becton-Dickinson FACS Canto, SanJose, CA).

### Tumor xenografts

The SKOV-NC and SKOV-miR-206 cells were harvested, washed twice with 1× PBS, and suspended in a serum-free medium. A suspension with 2.5×10^6^ cells in 0.2 ml serum-free medium was injected subcutaneously into the flank of 6-week-old BALB/c-nu/nu mice Orient Bio Inc. (Seongnam, South Korea). The tumor growth was monitored weekly by measuring two diameters of tumors with calipers for 8 weeks. The tumor volume was calculated by the formula V = (a^2^×b)/2, where a and b are the width and the length, respectively, of the tumor in millimeters. Each group contained four or five animals. This animal experiment was performed according to the institutional guidelines for the care and use of laboratory animals as adopted by the U.S. National Institutes of Health and the Catholic University Animal Care and Use Committee (approval number: 2016-015).

### Statistical analysis

Statistical significance was determined with Student’s unpaired t-tests or one-way analyses of variance, followed by Student-Newman-Keuls tests for multiple comparisons. These analyses were conducted with GraphPad Prism software (GraphPad Software Inc., La Jolla, CA).

## SUPPLEMENTARY MATERIALS FIGURES



## References

[1] Arteaga CL, Engelman JA (2014). ERBB receptors: from oncogene discovery to basic science to mechanism-based cancer therapeutics. Cancer Cell.

[2] Maroun CR, Rowlands T (2014). The Met receptor tyrosine kinase: a key player in oncogenesis and drug resistance. Pharmacol Ther.

[3] Sierra JR, Tsao MS (2011). c-MET as a potential therapeutic target and biomarker in cancer. Ther Adv Med Oncol.

[4] Ma PC, Tretiakova MS, MacKinnon AC, Ramnath N, Johnson C, Dietrich S, Seiwert T, Christensen JG, Jagadeeswaran R, Krausz T, Vokes EE, Husain AN, Salgia R (2008). Expression and mutational analysis of MET in human solid cancers. Genes Chromosomes Cancer.

[5] Natali PG, Prat M, Nicotra MR, Bigotti A, Olivero M, Comoglio PM, Di Renzo MF (1996). Overexpression of the met/HGF receptor in renal cell carcinomas. Int J Cancer.

[6] Yamamoto S, Tsuda H, Miyai K, Takano M, Tamai S, Matsubara O (2011). Gene amplification and protein overexpression of MET are common events in ovarian clear-cell adenocarcinoma: their roles in tumor progression and prognostication of the patient. Mod Pathol.

[7] Lu Z, Jiang G, Blume-Jensen P, Hunter T (2001). Epidermal growth factor-induced tumor cell invasion and metastasis initiated by dephosphorylation and downregulation of focal adhesion kinase. Mol Cell Biol.

[8] Geyer CE, Forster J, Lindquist D, Chan S, Romieu CG, Pienkowski T, Jagiello-Gruszfeld A, Crown J, Chan A, Kaufman B, Skarlos D, Campone M, Davidson N (2006). Lapatinib plus capecitabine for HER2-positive advanced breast cancer. N Engl J Med.

[9] Leo AD, Gomez H, Aziz Z, Zvirbule Z, Arbushites M, Oliva C, Koehler M, Williams L, Dering J, Finn R (2007). Lapatinib (L) with paclitaxel compared to paclitaxel as first-line treatment for patients with metastatic breast cancer: a phase III randomized, double-blind study of 580 patients. J Clin Oncol.

[10] Chen JT, Huang CY, Chiang YY, Chen WH, Chiou SH, Chen CY, Chow KC (2008). HGF increases cisplatin resistance via down-regulation of AIF in lung cancer cells. Am J Respir Cell Mol Biol.

[11] Tang MK, Zhou HY, Yam JW, Wong AS (2010). c-Met overexpression contributes to the acquired apoptotic resistance of nonadherent ovarian cancer cells through a cross talk mediated by phosphatidylinositol 3-kinase and extracellular signal-regulated kinase 1/2. Neoplasia.

[12] Ozasa H, Oguri T, Maeno K, Takakuwa O, Kunii E, Yagi Y, Uemura T, Kasai D, Miyazaki M, Niimi A (2014). Significance of c-MET overexpression in cytotoxic anticancer drug-resistant small-cell lung cancer cells. Cancer Sci.

[13] Engelman JA, Zejnullahu K, Mitsudomi T, Song Y, Hyland C, Park JO, Lindeman N, Gale CM, Zhao X, Christensen J, Kosaka T, Holmes AJ, Rogers AM (2007). MET amplification leads to gefitinib resistance in lung cancer by activating ERBB3 signaling. Science.

[14] Bean J, Brennan C, Shih JY, Riely G, Viale A, Wang L, Chitale D, Motoi N, Szoke J, Broderick S, Balak M, Chang WC, Yu CJ (2007). MET amplification occurs with or without T790M mutations in EGFR mutant lung tumors with acquired resistance to gefitinib or erlotinib. Proc Natl Acad Sci U S A.

[15] Yasuda H, Kobayashi S, Costa DB (2012). EGFR exon 20 insertion mutations in non-small-cell lung cancer: preclinical data and clinical implications. Lancet Oncol.

[16] Itoh K, Chiba T, Takahashi S, Ishii T, Igarashi K, Katoh Y, Oyake T, Hayashi N, Satoh K, Hatayama I, Yamamoto M, Nabeshima Y (1997). An Nrf2/small Maf heterodimer mediates the induction of phase II detoxifying enzyme genes through antioxidant response elements. Biochem Biophys Res Commun.

[17] Kwak MK, Wakabayashi N, Itoh K, Motohashi H, Yamamoto M, Kensler TW (2003). Modulation of gene expression by cancer chemopreventive dithiolethiones through the Keap1-Nrf2 pathway. Identification of novel gene clusters for cell survival. J Biol Chem.

[18] Itoh K, Wakabayashi N, Katoh Y, Ishii T, Igarashi K, Engel JD, Yamamoto M (1999). Keap1 represses nuclear activation of antioxidant responsive elements by Nrf2 through binding to the amino-terminal Neh2 domain. Genes Dev.

[19] McMahon M, Itoh K, Yamamoto M, Hayes JD (2003). Keap1-dependent proteasomal degradation of transcription factor Nrf2 contributes to the negative regulation of antioxidant response element-driven gene expression. J Biol Chem.

[20] Hayes JD, McMahon M, Chowdhry S, Dinkova-Kostova AT (2010). Cancer chemoprevention mechanisms mediated through the Keap1-Nrf2 pathway. Antioxid Redox Signal.

[21] O’Connell MA, Hayes JD (2015). The Keap1/Nrf2 pathway in health and disease: from the bench to the clinic. Biochem Soc Trans.

[22] Choi BH, Kwak MK (2016). Shadows of NRF2 in cancer: resistance to chemotherapy. Curr Opin Toxicol.

[23] Hayes JD, McMahon M (2009). NRF2 and KEAP1 mutations: permanent activation of an adaptive response in cancer. Trends Biochem Sci.

[24] Ji L, Li H, Gao P, Shang G, Zhang DD, Zhang N, Jiang T (2013). Nrf2 pathway regulates multidrug-resistance-associated protein 1 in small cell lung cancer. PLoS One.

[25] Singh A, Wu H, Zhang P, Happel C, Ma J, Biswal S (2010). Expression of ABCG2 (BCRP) is regulated by Nrf2 in cancer cells that confers side population and chemoresistance phenotype. Mol Cancer Ther.

[26] Ryoo IG, Kim G, Choi BH, Lee SH, Kwak MK (2016). Involvement of NRF2 signaling in doxorubicin resistance of cancer stem cell-enriched colonospheres. Biomol Ther (Seoul).

[27] Doyle LA, Yang W, Abruzzo LV, Krogmann T, Gao Y, Rishi AK, Ross DD (1998). A multidrug resistance transporter from human MCF-7 breast cancer cells. Proc Natl Acad Sci U S A.

[28] Litman T, Brangi M, Hudson E, Fetsch P, Abati A, Ross DD, Miyake K, Resau JH, Bates SE (2000). The multidrug-resistant phenotype associated with overexpression of the new ABC half-transporter, MXR (ABCG2). J Cell Sci.

[29] Doyle L, Ross DD (2003). Multidrug resistance mediated by the breast cancer resistance protein BCRP (ABCG2). Oncogene.

[30] An Y, Ongkeko WM (2009). ABCG2: the key to chemoresistance in cancer stem cells?. Expert Opin Drug Metab Toxicol.

[31] Mogi M, Yang J, Lambert JF, Colvin GA, Shiojima I, Skurk C, Summer R, Fine A, Quesenberry PJ, Walsh K (2003). Akt signaling regulates side population cell phenotype via Bcrp1 translocation. J Biol Chem.

[32] Hu C, Li H, Li J, Zhu Z, Yin S, Hao X, Yao M, Zheng S, Gu J (2008). Analysis of ABCG2 expression and side population identifies intrinsic drug efflux in the HCC cell line MHCC-97L and its modulation by Akt signaling. Carcinogenesis.

[33] Chen JS, Pardo FS, Wang-Rodriguez J, Chu TS, Lopez JP, Aguilera J, Altuna X, Weisman RA, Ongkeko WM (2006). EGFR regulates the side population in head and neck squamous cell carcinoma. Laryngoscope.

[34] Marchetti S, de Vries NA, Buckle T, Bolijn MJ, van Eijndhoven MA, Beijnen JH, Mazzanti R, van Tellingen O, Schellens JH (2008). Effect of the ATP-binding cassette drug transporters ABCB1, ABCG2, and ABCC2 on erlotinib hydrochloride (Tarceva) disposition *in vitro* and *in vivo* pharmacokinetic studies employing Bcrp1-/-/Mdr1a/1b-/- (triple-knockout) and wild-type mice. Mol Cancer Ther.

[35] Jung KA, Choi BH, Kwak MK (2015). The c-MET/PI3K signaling is associated with cancer resistance to doxorubicin and photodynamic therapy by elevating BCRP/ABCG2 expression. Mol Pharmacol.

[36] Papaiahgari S, Yerrapureddy A, Hassoun PM, Garcia JG, Birukov KG, Reddy SP (2007). EGFR-activated signaling and actin remodeling regulate cyclic stretch-induced NRF2-ARE activation. Am J Respir Cell Mol Biol.

[37] Yamadori T, Ishii Y, Homma S, Morishima Y, Kurishima K, Itoh K, Yamamoto M, Minami Y, Noguchi M, Hizawa N (2012). Molecular mechanisms for the regulation of Nrf2-mediated cell proliferation in non-small-cell lung cancers. Oncogene.

[38] Clavijo-Cornejo D, Enriquez-Cortina C, Lopez-Reyes A, Dominguez-Perez M, Nuno N, Dominguez-Meraz M, Bucio L, Souza V, Factor VM, Thorgeirsson SS, Gutierrez-Ruiz MC, Gomez-Quiroz LE (2013). Biphasic regulation of the NADPH oxidase by HGF/c-Met signaling pathway in primary mouse hepatocytes. Biochimie.

[39] Ciesla M, Marona P, Kozakowska M, Jez M, Seczynska M, Loboda A, Bukowska-Strakova K, Szade A, Walawender M, Kusior M, Stepniewski J, Szade K, Krist B (2016). Heme oxygenase-1 controls an HDAC4-miR-206 pathway of oxidative stress in rhabdomyosarcoma. Cancer Res.

[40] Meyer zu Schwabedissen HE, Grube M, Dreisbach A, Jedlitschky G, Meissner K, Linnemann K, Fusch C, Ritter CA, Volker U, Kroemer HK (2006). Epidermal growth factor-mediated activation of the map kinase cascade results in altered expression and function of ABCG2 (BCRP). Drug Metab Dispos.

[41] Huang WC, Chen YJ, Li LY, Wei YL, Hsu SC, Tsai SL, Chiu PC, Huang WP, Wang YN, Chen CH, Chang WC, Chang WC, Chen AJ (2011). Nuclear translocation of epidermal growth factor receptor by Akt-dependent phosphorylation enhances breast cancer-resistant protein expression in gefitinib-resistant cells. J Biol Chem.

[42] Dai CL, Tiwari AK, Wu CP, Su XD, Wang SR, Liu DG, Ashby CR, Huang Y, Robey RW, Liang YJ (2008). Lapatinib (Tykerb, GW572016) reverses multidrug resistance in cancer cells by inhibiting the activity of ATP-binding cassette subfamily B member 1 and G member 2. Cancer Res.

[43] Perry J, Ghazaly E, Kitromilidou C, McGrowder EH, Joel S, Powles T (2010). A synergistic interaction between lapatinib and chemotherapy agents in a panel of cell lines is due to the inhibition of the efflux pump BCRP. Mol Cancer Ther.

[44] Kim HK, Lee YS, Sivaprasad U, Malhotra A, Dutta A (2006). Muscle-specific microRNA miR-206 promotes muscle differentiation. J Cell Biol.

[45] Kondo N, Toyama T, Sugiura H, Fujii Y, Yamashita H (2008). miR-206 expression is down-regulated in estrogen receptor alpha-positive human breast cancer. Cancer Res.

[46] Wang XW, Xi XQ, Wu J, Wan YY, Hui HX, Cao XF (2015). MicroRNA-206 attenuates tumor proliferation and migration involving the downregulation of NOTCH3 in colorectal cancer. Oncol Rep.

[47] Yan D, Dong Xda E, Chen X, Wang L, Lu C, Wang J, Qu J, Tu L (2009). MicroRNA-1/206 targets c-Met and inhibits rhabdomyosarcoma development. J Biol Chem.

[48] Elliman SJ, Howley BV, Mehta DS, Fearnhead HO, Kemp DM, Barkley LR (2014). Selective repression of the oncogene cyclin D1 by the tumor suppressor miR-206 in cancers. Oncogenesis.

[49] Keklikoglou I, Hosaka K, Bender C, Bott A, Koerner C, Mitra D, Will R, Woerner A, Muenstermann E, Wilhelm H, Cao Y, Wiemann S (2015). MicroRNA-206 functions as a pleiotropic modulator of cell proliferation, invasion and lymphangiogenesis in pancreatic adenocarcinoma by targeting ANXA2 and KRAS genes. Oncogene.

[50] Chen QY, Jiao DM, Wang J, Hu H, Tang X, Chen J, Mou H, Lu W (2016). miR-206 regulates cisplatin resistance and EMT in human lung adenocarcinoma cells partly by targeting MET. Oncotarget.

[51] DeNicola GM, Karreth FA, Humpton TJ, Gopinathan A, Wei C, Frese K, Mangal D, Yu KH, Yeo CJ, Calhoun ES, Scrimieri F, Winter JM, Hruban RH (2011). Oncogene-induced Nrf2 transcription promotes ROS detoxification and tumorigenesis. Nature.

[52] Tao S, Wang S, Moghaddam SJ, Ooi A, Chapman E, Wong PK, Zhang DD (2014). Oncogenic KRAS confers chemoresistance by upregulating NRF2. Cancer Res.

[53] Papaiahgari S, Zhang Q, Kleeberger SR, Cho HY, Reddy SP (2006). Hyperoxia stimulates an Nrf2-ARE transcriptional response via ROS-EGFR-PI3K-Akt/ERK MAP kinase signaling in pulmonary epithelial cells. Antioxid Redox Signal.

[54] Singh A, Happel C, Manna SK, Acquaah-Mensah G, Carrerero J, Kumar S, Nasipuri P, Krausz KW, Wakabayashi N, Dewi R, Boros LG, Gonzalez FJ, Gabrielson E (2013). Transcription factor NRF2 regulates miR-1 and miR-206 to drive tumorigenesis. J Clin Invest.

[55] Choi BH, Ryoo IG, Kang HC, Kwak MK (2014). The sensitivity of cancer cells to pheophorbide a-based photodynamic therapy is enhanced by Nrf2 silencing. PLoS One.

[56] Manandhar S, Choi BH, Jung KA, Ryoo IG, Song M, Kang SJ, Choi HG, Kim JA, Park PH, Kwak MK (2012). NRF2 inhibition represses ErbB2 signaling in ovarian carcinoma cells: implications for tumor growth retardation and docetaxel sensitivity. Free Radic Biol Med.

[57] Jung KA, Lee S, Kwak MK (2017). NFE2L2/NRF2 activity is linked to mitochondria and AMP-activated protein kinase signaling in cancers through miR-181c/mitochondria-encoded cytochrome c oxidase regulation. Antioxid Redox Signal.

[58] Ryoo IG, Shin DH, Kang KS, Kwak MK (2015). Involvement of Nrf2-GSH signaling in TGFbeta1-stimulated epithelial-to-mesenchymal transition changes in rat renal tubular cells. Arch Pharm Res.

