# Supplementary material for: NFE2L2/NRF2 silencing-inducible miR-206 targets c-MET/EGFR and suppresses BCRP/ABCG2 in cancer cells
Source: Oncotarget. 2017 Nov 18;8(63):107188–205. doi: 10.18632/oncotarget.22513 (PMC5739807; doi:10.18632/oncotarget.22513)
Supplement: Supplementary file 1 [file oncotarget-08-107188-s001.pdf]

NFE2L2/NRF2 silencing-inducible miR-206 targets c-MET/EGFR and suppresses BCRP/ABCG in cancer cells

SUPPLEMENTARY MATERIALS

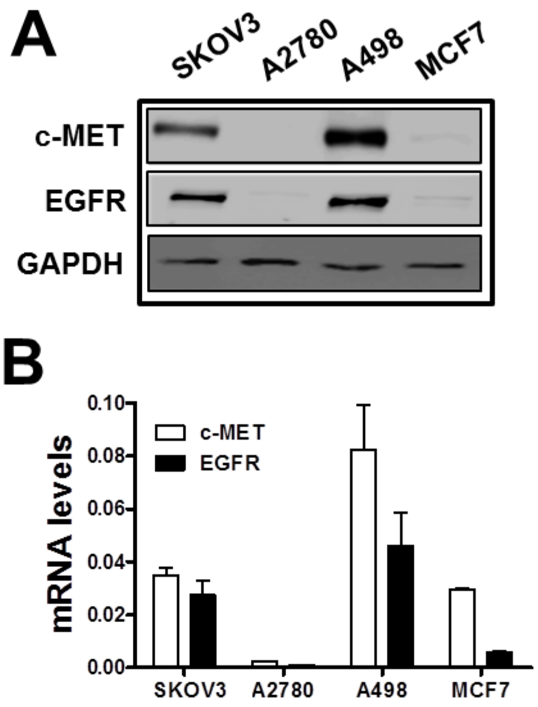

Supplementary Figure 1: (A) Protein levels for c-MET and EGFR in four cell lines: ovarian carcinoma SKOV3 and A2780, renal carcinoma A498, and breast carcinoma MCF7. **(B)** Transcript levels for c-MET and EGFR were assessed in four cell lines. Transcript levels of each gene were normalized with the level of HPRT.

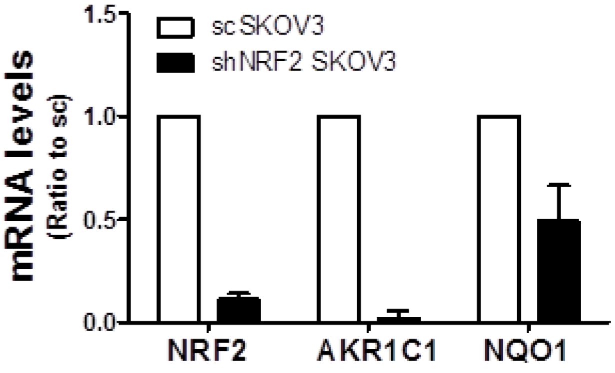

Supplementary Figure 2: ***NRF2*-silending was verified by measuring transcript levels for *NRF2*, *AKR1C1* and *NQO-1* in established scSKOV3 and shNRF2-SKOV3 cells.** Values are means ± SD from 3-4 experiments.

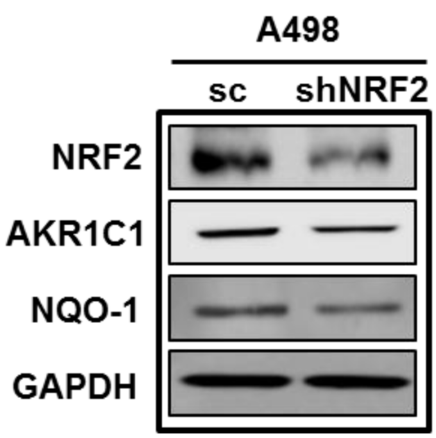

Supplementary Figure 3: NRF2, AKR1C1, and NQO-1 protein levels were determined by Western blotting in scA498 and shNRF2-A498 cells.

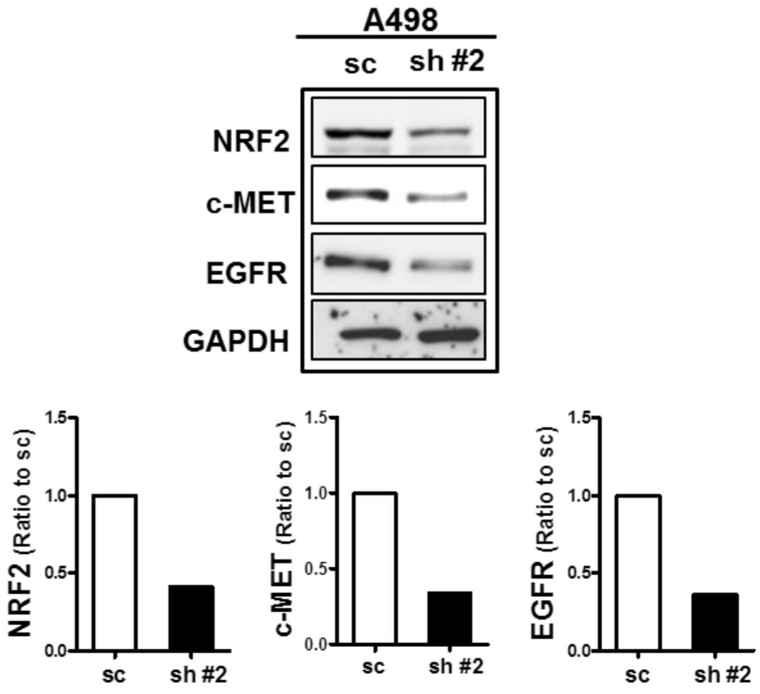

Supplementary Figure 4: Additional *NRF2*-specific shRNA (shNRF2 #2) was transiently transduced into A498, and protein levels of NRF2, c-MET and EGFR were assessed.

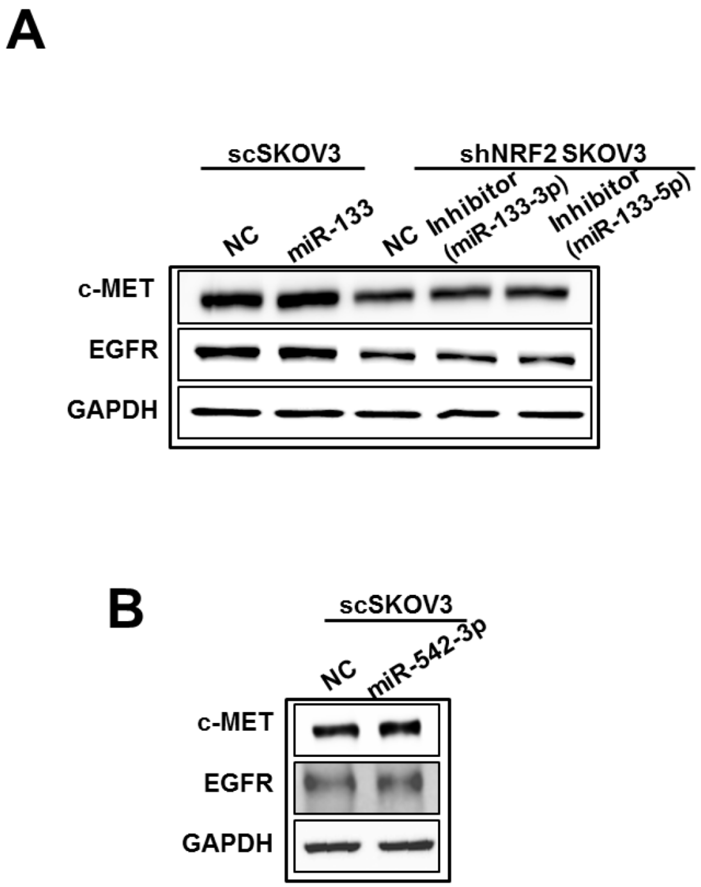

Supplementary Figure 5: (A) The effect of the miR-133 mimic or miR-133 inhibitor on c-MET and EGFR protein levels. The scSKOV3 cells were transfected with the miR-133 mimic (100 nM) and the shNRF2 cells were transfected with the miR-133 inhibitor (100 nM). After 18 h-incubation, protein levels for c-MET and EGFR were monitored by Western blotting. **(B)** After transfection of the scSKOV3 with the miR-542 mimic (100 nM), c-MET and EGFR protein level was measured.

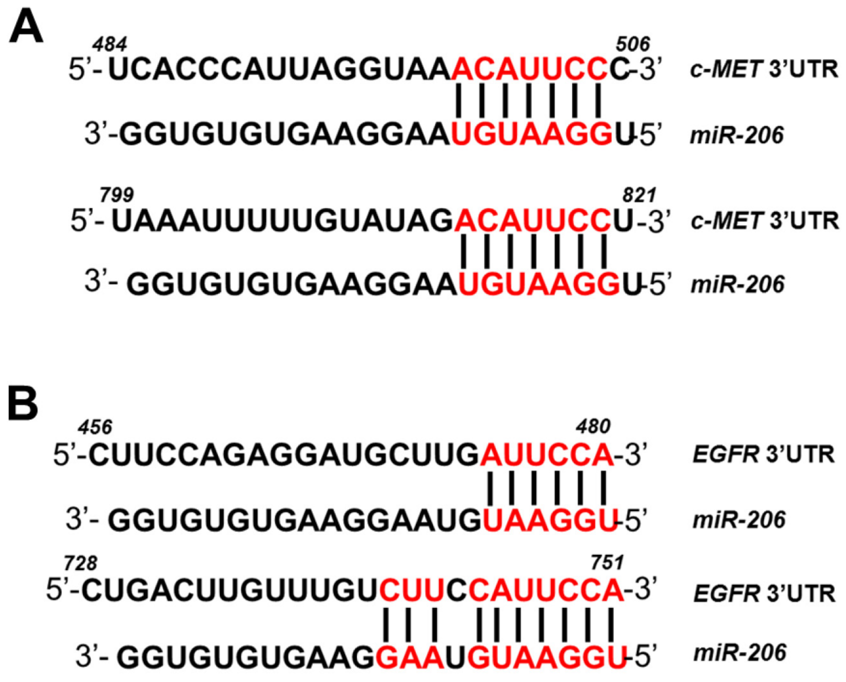

Supplementary Figure 6: Target analysis with TargetScan (http://www.targetscan.org/vert_71/) and Diana Tools (http://diana.imis.athena-innovation.gr/DianaTools/index.php?r=tarbase/index) showed that miR-206 can bind to the 3'-UTR of the *c-MET* **(A)** and *EGFR* **(B)** gene.

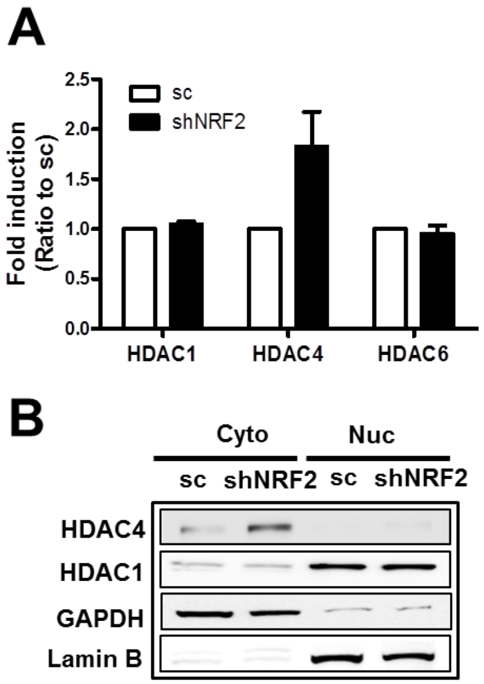

Supplementary Figure 7: (A) The transcript levels for HDAC1, HDAC4, and HDAC6 were measured in sc and shNRF2-SKOV3 cells. **(B)** Cytoplasmic (Cyto) and nuclear (Nuc) protein levels of HDAC1 and HDAC4 were assessed by Western blotting.
